# Self-Selection of Feeding Substrates by *Tenebrio molitor* Larvae of Different Ages to Determine Optimal Macronutrient Intake and the Influence on Larval Growth and Protein Content

**DOI:** 10.3390/insects13070657

**Published:** 2022-07-21

**Authors:** Nina Kröncke, Rainer Benning

**Affiliations:** Institute of Food Technology and Bioprocess Engineering, University of Applied Sciences Bremerhaven, An der Karlstadt 8, 27568 Bremerhaven, Germany; rbenning@hs-bremerhaven.de

**Keywords:** *Tenebrio molitor*, insect rearing, nutrient balance, self-selection, nutritional composition, insect diet, macronutrients

## Abstract

**Simple Summary:**

Insects are widely discussed as being an alternative source of protein and a substitute for soy- and fishmeal. However, mass production is still expensive, so means are being sought to reduce production costs. One way is to keep the costs for insect diets low. One such possibility could be the use of food industry side streams or agricultural by-products to formulate an insect diet. This study tested different substrates as feed for mealworm larvae. These included by-products from grain processing such as husks or bran, pomace and other plant components. In many studies, feed is often tested individually, during which insect larvae are given a defined substrate as a single feed or in a two-component mixture, which they either eat or reject. It is often difficult to define the right nutrient composition for creating an insect diet. Therefore, the method of self-selection was used, in order to draw conclusions about the necessary nutrient requirements, especially depending on the age of the larvae. The results showed that the most suitable substrates for rearing mealworm larvae were mainly wheat bran and flour, maize hulls, oat bran and flakes, rice flour, lupine flour and potato flakes. Irrespective of their age, the larvae required a high content of carbohydrates and proteins and a low amount of fats and minerals. Indeed, protein and fat requirements grow with increasing age. These findings contribute towards formulating diet mixtures for *Tenebrio molitor* larvae of different ages.

**Abstract:**

Nutrient self-selection was used to determine the optimal uptake of macronutrients by the yellow mealworm (*Tenebrio molitor*) larvae. The selection study consisted of four combinations of eight pelleted substrates from a total choice of 25, available to the larvae in a multiple-choice arena. In order to be able to determine the nutrient requirements as a function of the larvae age, six, eight and tenweekold larvae were used for the experiment. The larvae were free to choose between the different feeds for a period of two weeks. Rearing took place at 27 °C, 75% relative humidity and under dark conditions. The optimal ratios of macronutrients were 67.3 to 71.5% for carbohydrates, 19.9 to 22.8% for proteins and 8.6 to 10.0% for lipids to ensure the best results. Biomass growth, food intake and conversion were positively influenced to a significant extent by carbohydrate intake. The protein content, too, varied according to the macronutrient intake and substrate composition; a higher protein consumption increased the larval protein content. Wheat bran and flour, oat bran and flakes, maize hulls, lupine flour and potato flakes, in particular, were considered suitable substrates for the feeding and rearing of *Tenebrio molitor* larvae and highlighted that these larvae preferred a grain-based diet.

## 1. Introduction

The yellow mealworm (*Tenebrio molitor* L., Coleoptera: Tenebrionidae) is an important species for rearing industrial insects in the European market. In recent years, there has been increased consumer interest in more sustainable food and protein sources for livestock feeding. The larvae of *Tenebrio molitor* have been shown to be an acceptable protein source for broiler chickens and can be used to completely replace soybean meal in broiler diets [1]. However, high labor costs associated with insect rearing and high prices for larval diet formulations have resulted in the market price of insect products becoming more costly than other protein-rich food products, meaning that insect products have, thus, only been competitive in the ‘novel’ food sector. For mass insect production, it is imperative that the insect diet is inexpensive and effective, in order to minimize production costs and maximize biomass productivity. From an ecological point of view, an ideal insect diet would consist of waste products or plant-based agricultural by-products that do not compete with human nutrition or are unsuitable for consumption [2].

Insects that have different substrates at their disposal usually choose to balance the required nutrients such as carbohydrates, proteins and fats [3]. Herbivorous insects tightly regulate their nutrient intake when given the opportunity to adjust their growth, reproduction and development [4]. The balancing of the protein and carbohydrate content, with incomplete nutrition, was proven in locusts (*Locusta migratoria* L., Orthoptera: Acrididae) [5]. Different studies have examined the use of agricultural by-products [6,7,8,9,10,11,12], fish discards [13] and organic waste [14] as insect food and the effects on larval chemical composition. Researchers concluded that it is possible to rear mealworm larvae on food by-products. However, feed conversion efficiency, development time and nutritional value are strongly influenced by substrate composition. Indeed, the focus is often only on individual substrates and their impact on the utilization and growth parameters. The formulation of insect diets is complex, so that suitable food formulations can only be found by providing the larvae with several components featuring different nutritional values, which the animals can freely choose through self-selection. 

The nutritional requirements of mealworm larvae, particularly amino acids and protein, have been studied [15,16,17]. There are already some studies by Morales-Ramos [18,19,20] based on the self-selection method of Waldbauer and Friedman [3]. These studies reported on the ability of mealworm larvae to regulate their intake of macronutrients and demonstrate that dietary self-selection appears in this insect species. Dietary self-selection was also observed in *Tribolium confusum* (Herbst), which belongs to the Tenebrionidae family and has comparable feeding habits to *Tenebrio molitor*. 

The aims of this study were: (1) to determine the optimal macronutrient ratios of *Tenebrio molitor* larvae, based on the self-selection of 25 various substrates with different nutrient content, (2) to establish suitable substrates for feeding mealworm larvae and the formulation of insect diets, (3) to study the effect of feed on biomass growth, food intake, utilization efficiency and protein content, and (4) to see whether the larvae have different nutrient requirements depending on their age. Understanding the macronutrient requirements could improve the development of optimal feed mixtures that advance the use of *Tenebrio molitor* for insect mass rearing. 

## 2. Materials and Methods

### 2.1. Insect Samples

Mealworm larvae used in this study were reared at the University of Applied Sciences Bremerhaven in a constant climate chamber (HPP 110, Memmert, Schwabach, Germany) at 27 °C with a relative humidity of 75% and were fed with wheat bran ad libitum until larvae were selected for the experiment. Larvae of different ages (six-, eight- and ten-weeks-old) were used for this study. The experimental groups consisted of 100 larvae, which were placed in multiple-choice arenas. All larvae were weighed at the beginning and end of the experiment, and their biomass was recorded. The larvae were sorted as well at the beginning of the experiment according to a consistent size with an average length of 8.5 ± 0.2 mm (six-weeks-old), 11.4 ± 0.2 mm (eight-weeks-old) and 14.6 ± 0.1 mm (ten-weeks-old), with the result that very large or small larvae were not used for the experiment to ensure that the larvae reached a similar instar in the same aging group. According to the larval weight, size and development time, the larvae reached instar 6–7 (six-weeks-old), 8–9 (eight-weeks-old) and 10–11 (ten-weeks-old), respectively [21,22]. After an experimental period of two weeks, larvae were separated from uneaten feed and frass using a sieve. After data collection, larvae were starved for 24 h, frozen at −21 °C for 48 h in a freezer (HAS 47520, Beko, Neu-Isenburg, Germany) and stored, before protein content analyses were carried out. Residue weight was included to calculate food conversion efficiency. Dead larvae were counted at the end of the investigation to determine the survival rate.

### 2.2. Experimental Multiple-Choice Arenas

**Figure 1:**
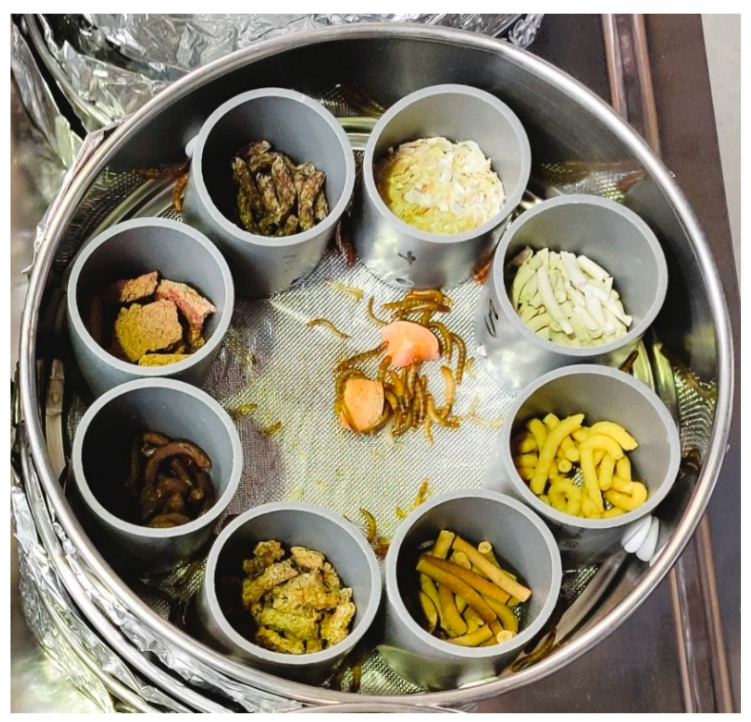
Multiple-choice arena for self-selection, comprising eight different substrates inside a plastic tube, with larvae and carrots in the center.

The multiple-choice arenas (Figure 1) consisted of flour sieves (diameter 160 mm and height 50 mm, Karl Weis u. Cie. GmbH, Murr, Germany) with a mesh size of 0.64 mm. Eight plastic tubes (diameter and height 40 mm, OBI GmbH & Co. KG, Wermelskirchen, Germany) were placed on the sieve bottom and attached to the side of the sieve with a small dot of adhesive (Patafix, UHU GmbH & Co. KG, Bühl/Baden, Germany). The tubes were arranged radially, equidistant from the center of the sieve. A small opening (diameter 5 mm and height 3 mm from the sieve bottom) was drilled in each tube with a pillar drill (TE-BD 750 E, Einhell Germany AG, Landau/Isar, Germany) so that the larvae could freely enter the tubes. Aluminum foil was attached to the bottom of the sieve so that the frass could be caught and weighed at the end of the experiment.

### 2.3. Feeding Treatments

Four different feeding treatments with eight various ingredients, consisting of 25 substrates, were tested (Table 1). Substrates were chosen because of their differences in nutritional composition as specified by the manufacturer (see Table S1 in Supplementary Material). The combination of the treatments was based on the content of macronutrients, especially protein, carbohydrates, lipids and fiber. Every treatment contained a substrate with a high content of each of the macronutrients, so that the mealworm larvae could choose a complete diet according to their needs and their age.

Substrates were ground into powder using a spice grinder (EGK 200, Rommelsbacher ElektroHausgeräte GmbH, Dinkelsbühl, Germany) and mixed with different ratios of water (40 to 70%) to obtain a dough-like consistency. Short, thin strips were formed using a 50 mL syringe and then dried in an oven (UT 6120, Heraeus Instruments, Hanau, Germany) at 60 °C for 21 h. The dry sticks were then placed in the plastic tubes to create multiple-choice arenas (Figure 1). Each treatment consisted of three repetitions (=three test units with a total of 300 larvae). The substrates were randomly distributed in the tubes to minimize proximity effects between the different substrates. At the beginning of the experiment, a measured amount (3 g) of each substrate was placed in the appropriate arena compartment in each of the experimental units. The amount fed was recorded for each of the experimental units of each of the treatment combinations. In addition, 3 g of carrots were given each week and placed in the center of the arena to provide the larvae with water. The experiment was monitored daily to observe consumption. Substrates depleted by consumption were replenished.

Larvae of different ages (six-, eight- and ten-weeks-old) were used for the study and fed with the four self-selection feeding treatments, which were repeated three times per group (Table 2). 

### 2.4. Calculations

Larval weight, survival rate and the amount of uneaten feed were recorded at the end of the experiment. Larval weight gain per larva, specific growth rate and the efficiency of conversion of ingested food were subsequently calculated as described by Waldbauer (1968) [23]. Data for weight gained per larva (LWGpL; Equation (1)) were used to determine feed utilization parameters

(1) $$\mathrm{LWGpL}=\frac{\mathrm{Larval}\;\mathrm{weight}\;\mathrm{end}\;-\;\mathrm{Larval}\;\mathrm{weight}\;\mathrm{start}\;}{\mathrm{Number}\;\mathrm{of}\;\mathrm{larvae}\;\mathrm{at}\;\mathrm{beginning}\;-\;\mathrm{Number}\;\mathrm{of}\;\mathrm{dead}\;\mathrm{larvae}}$$

The specific growth rate (SGR, % per day; Equation (2)) was calculated according to the following formula, where FBW stands for final body weight and IBW for initial body weight

(2) $$\mathrm{SGR}=\frac{\mathrm{lnFBW}-\;\mathrm{lnIBW}}{\mathrm{experimental}\;\mathrm{days}}\;\times \;100\%$$

as well as the efficiency of conversion of ingested food (ECI, %; Equation (3))

(3) $$\mathrm{ECI}=\frac{\mathrm{Weight}\;\mathrm{gained}}{\mathrm{Feed}\;\mathrm{consumed}}\;\times \;100\%$$

Macronutrient intake was calculated as described by Morales-Ramos (2020) [24] by dividing carbohydrate, protein and lipid content by the sum of all macronutrients (carbohydrates + protein + lipids = 100%) in the ingredients of each substrate. In addition, the intake of all nutrients was calculated (carbohydrates + protein + lipids + fiber + ash) to examine the effects of fiber and minerals.

### 2.5. Protein Content Analysis

Protein content (% of fresh weight basis) of mealworm larvae was measured using the Kjeldahl method and was calculated according to DIN EN 25663 and the Association of German Agricultural Analytic and Research Institutes by multiplying the measured nitrogen content by a factor of 6.25 [25].

### 2.6. Data Analysis

Statistical significance of the feeding results and protein content were replicated and performed three times (*n* = 3) and checked for normality (Shapiro–Wilk test) and homogeneity of variances (Bartlett’s test). All percentage data were transformed prior to analysis (arcsine square root transformation). Statistical analysis was performed by one-way ANOVA, followed by Tukey–Kramer post hoc test using SigmaPlot 12.5 (Systat Software Inc., Düsseldorf, Germany). A confidence interval of 95% (*p* < 0.05) was presumed.

## 3. Results

### 3.1. Macronutrient Intake

**Figure 2:**
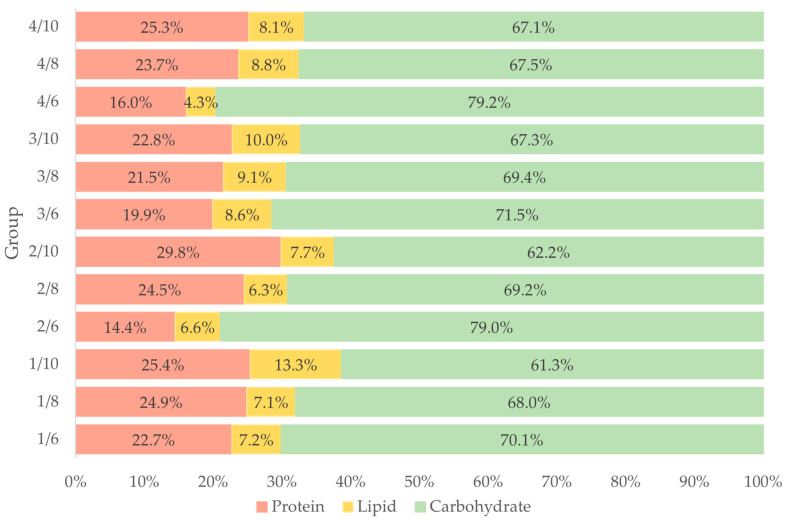
Percentage of consumed macronutrients (carbohydrates, protein, lipids) of four feeding groups (1–4) of *Tenebrio molitor* larvae at different larval ages (six-, eight- and ten-weeks-old); 1/6, 2/6, 3/6, 4/6: six-week-old larvae fed with treatment 1, 2, 3 and 4; 1/8, 2/8, 3/8, 4/8: eight-week-old larvae fed with treatment 1, 2, 3 and 4; 1/10, 2/10, 3/10, 4/10: ten-week-old larvae fed with treatment 1, 2, 3 and 4.

A wide variability was observed in the relative consumption of macronutrients. Macronutrient intake varied between 0.40 and 4.42 g/100 g for carbohydrates (C), 0.14 and 2.63 g/100 g for protein (P) and 0.05 and 0.34 g/100 g for lipids (L) (Table 3). The percentage intake of macronutrients (Figure 2) shows that younger larvae—aged six weeks—consumed more carbohydrates (70.1 to 79.2%) but fewer proteins (14.4 to 22.7%) and lipids (4.3 to 7.2%) than larvae aged eight weeks (67.5 to 69.4% C, 21.5 to 24.9% P, 6.3 to 9.1% L) or ten weeks (61.3 to 67.1% C, 22.8 to 29.8% P, 7.7 to 13.3% L). In general, the requirement for carbohydrates decreases with age, with a concomitant increased need for proteins and lipids.

**Figure 3:**
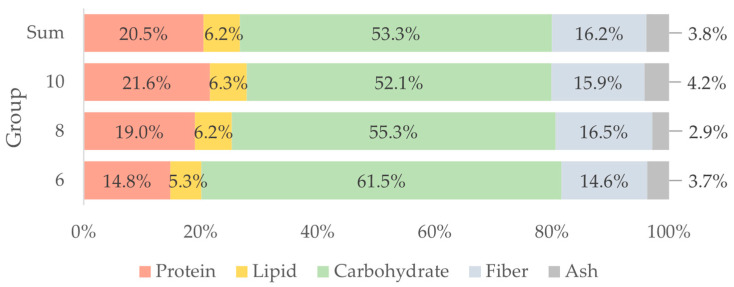
Percentage of consumed nutrients (carbohydrates, protein, lipids, fiber, ash) of *Tenebrio molitor* larvae at different larval ages (six-, eight- and ten-weeks-old) and the sum of all groups.

The nutrient intake of all nutritional ingredients (in mg/100 g) of mealworm larvae at different ages compared to the sum of all groups are presented in Table 4. Carbohydrates are still the main component of the diet in all groups (52.1 to 61.5%), followed by proteins (14.8 to 21.6%) (Figure 3). When all nutrients are related, it is noticeable that the larvae also ingest a high content of fiber (14.6 to 16.5%) in their diet, almost comparable to the protein content. The consumption of minerals (2.9 to 4.2%) is the smallest part of the diet. Independent of larval age, *Tenebrio molitor* larvae need a high amount of carbohydrates and proteins and fewer fibers, lipids and minerals.

**Figure 4:**
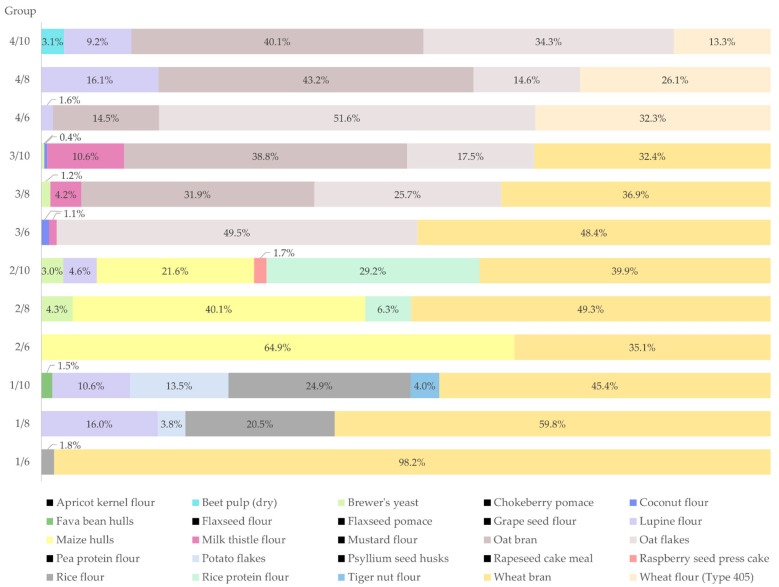
Percentage of consumed substrates from four feeding groups of *Tenebrio molitor* larvae at different larval ages (six-, eight-, ten-weeks-old); 1/6, 2/6, 3/6, 4/6: six-week-old larvae fed with treatment 1, 2, 3 and 4; 1/8, 2/8, 3/8, 4/8: eight-week-old larvae fed with treatment 1, 2, 3 and 4; 1/10, 2/10, 3/10, 4/10: ten-week-old larvae fed with treatment 1, 2, 3 and 4.

The consumption average of each substrate within the treatment combinations is shown in Supplementary Material (Table S2), and the consumption of each ingredient as a percentage is presented in Figure 4. Higher amounts of substrates consumed included wheat bran (32.4 to 98.2%), maize hulls (21.6 to 64.9%), oat flakes (14.6 to 51.6%), oat bran (31.9 to 43.2%), wheat flour (13.3 to 32.3%), rice protein flour (6.3 to 29.2%), rice flour (1.8 to 24.9%), lupine flour (1.6 to 16.1%) and potato flakes (3.8 to 13.5%). Substrates consumed the least were milk thistle flour (1.1 to 10.6%), tiger nut flour (4.0%), brewer’s yeast (0.4 to 4.3%), dried beet pulp (3.1%), raspberry seed press cake (1.7%), fava bean hulls (1.5%) and coconut flour (0.4 to 1.1%). Substrates not consumed at all were apricot kernel flour, chokeberry pomace, flaxseed flour and pomace, grape seed flour, mustard flour, pea protein flour, psyllium seed husks and rapeseed cake meal. Ingredients that had a high consumption are generally high in carbohydrate content. Low percentages of consumption or no consumption are based on substrates featuring a high amount of fiber or protein.

### 3.2. Growth Parameters

**Figure 5:**
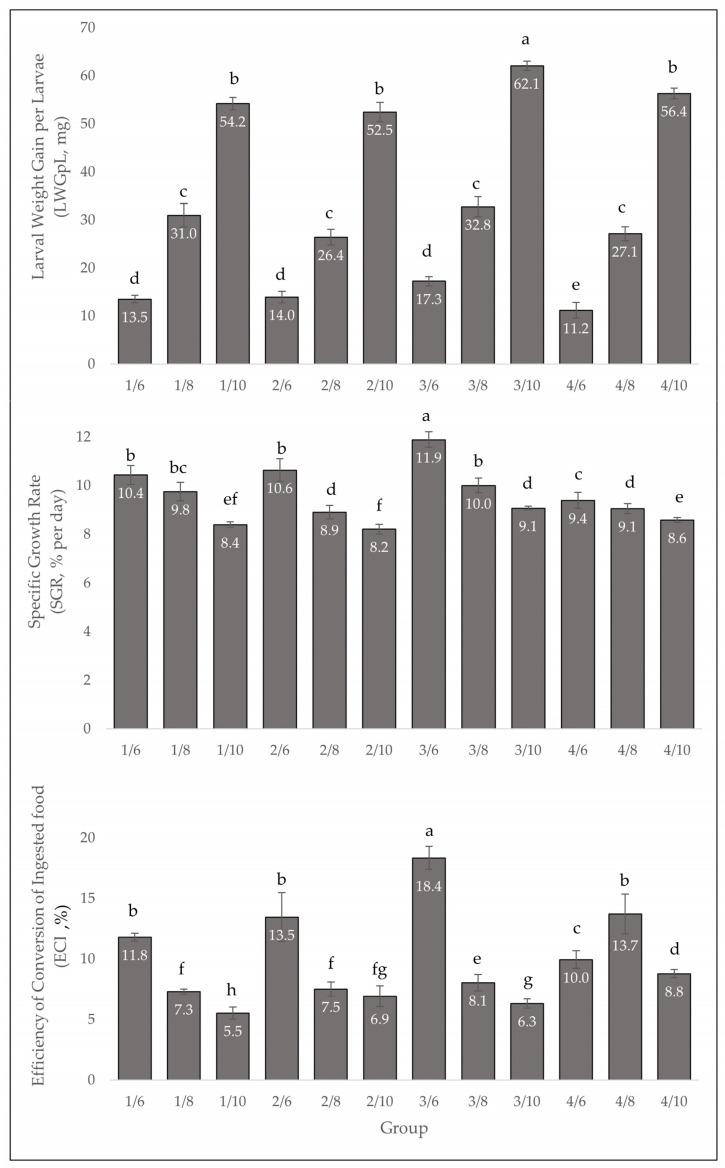
Larval weight gain per larva (LWGpL, mg), specific growth rate (SGR, % per day) and efficiency of conversion of ingested food (ECI, %) of *Tenebrio molitor* larvae at different larval ages (six-, eight-, ten-weeks-old) from four feeding groups, mean ± standard deviation (*n* = 3), different letters denote significant differences, 1/6, 2/6, 3/6, 4/6: six-week-old larvae fed with treatment 1, 2, 3 and 4; 1/8, 2/8, 3/8, 4/8: eight-week-old larvae fed with treatment 1, 2, 3 and 4; 1/10, 2/10, 3/10, 4/10: ten-week-old larvae fed with treatment 1, 2, 3 and 4.

Larval weight gain per larva (LWGpL in mg), specific growth rate (SGR in % per day) and food conversion efficiency (ECI in %) differ significantly with age (Figure 5). Younger larvae have a significantly higher growth rate (*F* = 11.85; df = 3.25; *p* = 0.001) and are more efficient at converting food than older larvae. In addition, the consumption of some substrates has a significant impact on food intake, biomass gain and ECI, especially in treatment 3. The survival rate of the groups was between 97.3 and 100%, with no significant differences (*F* = 1.78; df = 4.45; *p* = 0.643.)

### 3.3. Protein Content

**Figure 6:**
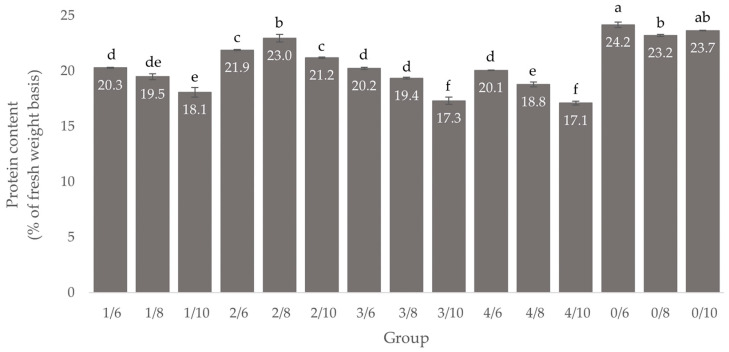
Protein content (% of fresh weight basis, mean ± standard deviation, *n* = 3) of *Tenebrio molitor* larvae at different larval ages (six-, eight-, ten-weeks-old) from four feeding groups and at the start of the experiment (Group 0), different letters denote significant differences, 1/6, 2/6, 3/6, 4/6: six-week-old larvae fed with treatment 1, 2, 3 and 4; 1/8, 2/8, 3/8, 4/8: eight-week-old larvae fed with treatment 1, 2, 3 and 4; 1/10, 2/10, 3/10, 4/10: ten-week-old larvae fed with treatment 1, 2, 3 and 4.

Protein content (% of fresh weight basis) of *Tenebrio molitor* larvae reared on four feeding groups with eight different substrates, as well as the larvae at the start of the experiment (Group 0), are shown in Figure 6. At the beginning of the experiment, larvae had the highest protein content; this decreased with age. Only the larvae in the experimental feeding group 2/8 had a higher protein content compared to the larvae at the beginning of the experiment and differed significantly (*F* = 8.47; df = 2.88; *p* = 0.001) to the other groups. In general, protein content decreases as the larval age increases, except in groups 2/8 and 0/10.

## 4. Discussion

The results presented in this study are consistent with previous studies and show that *Tenebrio molitor* larvae tend to regulate their uptake of nutrients by choosing from a variety of substrates during feeding and have a self-selection ability to ensure an optimal macronutrient ratio [18,19,20,26,27]. The intake of macronutrients varied greatly according to the specific feed. Excessive protein intake reduced the amount of carbohydrates consumed as well as the food conversion and larvae growth rate. The optimal ratio of macronutrients for mealworm larvae could be that which performed best in treatment 3 with 8.6 to 10.0% for lipids, 19.9 to 22.8% for proteins and 67.3 to 71.5% for carbohydrates. Morales-Ramos et al., (2020) [19] observed a similar ratio of carbohydrates, proteins and lipids that produced the best growth performance for *Tenebrio molitor* larvae. However, younger larvae need more carbohydrates for growth than older ones. Protein and lipid consumption increases with age. This may be explained by the fact that the larvae are preparing for metamorphosis, which is energy intensive [28], meaning that proteins and primary lipids are used as an energy supplier.

The larvae in treatment 2 had the highest protein content. These larvae consumed protein-rich substrates such as brewer’s yeast, so that a higher protein consumption and content of the feed resulted in an increasing protein content of the larvae. This was also observed by Rumbos et al., (2020) [29]. Compared to the six-week-old larvae at the beginning of the experiment, all larvae recorded a lower protein content at the end of the study. Older larvae (at ten weeks) showed a significant decrease in protein content compared to six- and eight-week-old larvae, except in treatment 2. This can be explained by a shift in protein and lipid content since *T. molitor* larvae accumulate more lipids as they increase in age [30].

Mealworm larvae are capable of consuming a wide range of organic waste and materials [31], so other potential feedstocks and plant-based by-products were investigated in this study. Rearing mealworm larvae typically uses wheat bran, wheat flour and the addition of brewer’s yeast and a plant-based water source such as carrots to provide optimal nutrition [32]. The perfect insect diet for *T. molitor* larvae is grain-based, containing ideally a mixture of grain and raw fresh vegetables [15]. Edible insects may have different prices, depending on the given substrate and the current price of it. By-products from the food industry or grain processing can usually be obtained inexpensively and are therefore an interesting source for insect nutrition [33].

Substrates showing an average consumption above 10% were considered suitable for feeding *Tenebrio molitor*. Relevant ingredients that had a positive impact on biomass growth included wheat bran and flour, oat bran and flakes, corn husk flour and rice protein flour and rice flour, lupine flour and potato flakes. These substrates have also been considered acceptable for feeding mealworm larvae in other studies [19,29]. Fava bean hulls, coconut flour, brewer’s yeast, raspberry seed cake, tiger nut powder and dried sugar beet pulp were consumed in small amounts and are therefore suitable as a diet, but should only be added in small amounts to the diet of *T. molitor*. Substrates that were not eaten may contain ingredients that have an adverse effect on growth rate due to the imbalance in macronutrients [19], such as psyllium husks. These have a very high fiber content [34] or are harmful to insects, such as essential oils in mustard flour that can act as an insecticide [35].

## 5. Conclusions

Agricultural by-products can offer suitable nutrition for *Tenebrio molitor* larvae, provided they are given in the right ratios and combinations. Relevant ingredients for feeding *T. molitor* larvae include wheat bran and flour, maize hulls, oat bran and flour, rice protein and flour, lupine flour and potato flakes. This clearly indicates that mealworm larvae prefer a diet high in grain. Self-selection is an effective method to approximate optimal combination ratios of substrates for mealworm larvae. The ratios and combinations of preferred substrates had a significant impact on larval growth, the increase in biomass and feed conversion. It seems that the most important factor affecting the weight gain of *Tenebrio molitor* larvae is the carbohydrate and protein content of the substrate. However, if the observed self-selected nutrient intake ratios deviate, insufficient or excessive intake of these nutrients could have an adverse impact on the feed conversion and larval growth of mealworm larvae. The protein content of the larvae is also influenced by substrate composition and macronutrient uptake since a higher protein content in the diet can increase the protein content of *Tenebrio molitor* larvae, thereby accentuating this insect as an attractive alternative source of protein.

## Figures and Tables

**Table 1:** Combination of eight different substrates (selection marked with an X) consisting of 25 substrates in four feeding treatments (containing a high content of each macronutrient) given to mealworm larvae for self-selection.

Substrate	Treatment			
	1	2	3	4
Apricot kernel flour				X
Beet pulp (dry)				X
Brewer’s yeast		X	X	
Chokeberry pomace				X
Coconut flour			X	
Fava bean hulls	X			
Flaxseed flour		X		
Flaxseed pomace				X
Grape seed flour		X		
Lupine flour	X	X		X
Maize hulls		X		
Milk thistle flour			X	
Mustard flour			X	
Oat bran			X	X
Oat flakes			X	X
Pea protein flour	X			
Potato flakes	X			
Psyllium seed husks			X	
Rapeseed cake meal	X			
Raspberry seed press cake		X		
Rice flour	X			
Rice protein flour		X		
Tiger nut flour	X			
Wheat bran	X	X	X	
Wheat flour				X

**Table 2:** Feeding treatments (three repetitions per group) of *Tenebrio molitor* larvae at different ages (six--, eight- and ten-weeks-old).

Group	Treatment	Larval Age (in Weeks)
1/6	1	6
1/8	1	8
1/10	1	10
2/6	2	6
2/8	2	8
2/10	2	10
3/6	3	6
3/8	3	8
3/10	3	10
4/6	4	6
4/8	4	8
4/10	4	10

**Table 3:** Macronutrient (carbohydrates, protein and lipids) intake (in g/100 g) of mealworm larvae in four self-selection treatments with various combinations of eight different substrates; mean ± standard deviation.

Group	Carbohydrates (g/100 g)	Protein (g/100 g)	Lipids (g/100 g)
1/6	0.52 ± 0.05 ^f^	0.17 ± 0.01 ^f^	0.05 ± 0.00 ^f^
1/8	2.00 ± 0.09 ^d^	0.73 ± 0.14 ^d^	0.21 ± 0.04 ^d^
1/10	0.40 ± 0.16 ^g^	0.17 ± 0.42 ^f^	0.09 ± 0.14 ^e^
2/6	0.63 ± 0.12 ^f^	0.12 ± 0.04 ^f^	0.05 ± 0.01 ^f^
2/8	1.74 ± 0.17 ^d^	0.62 ± 0.15 ^d^	0.16 ± 0.02 ^e^
2/10	2.79 ± 0.34 ^c^	2.63 ± 0.16 ^a^	0.34 ± 0.05 ^c^
3/6	0.48 ± 0.04 ^g^	0.14 ± 0.01 ^f^	0.06 ± 0.00 ^f^
3/8	1.93 ± 0.13 ^d^	0.60 ± 0.03 ^d^	0.25 ± 0.01 ^d^
3/10	4.42 ± 0.47 ^a^	1.50 ± 0.07 ^b^	0.65 ± 0.04 ^a^
4/6	0.72 ± 0.01 ^f^	0.14 ± 0.01 ^f^	0.04 ± 0.01 ^g^
4/8	1.01 ± 0.15 ^e^	0.35 ± 0.06 ^e^	0.13 ± 0.04 ^e^
4/10	3.35 ± 0.16 ^b^	1.02 ± 0.05 ^c^	0.44 ± 0.03 ^b^

^a–g^ Different superscripts for every column denote significant differences; *p* < 0.05; 1/6, 2/6, 3/6, 4/6: six-week-old larvae fed with treatment 1, 2, 3 and 4; 1/8, 2/8, 3/8, 4/8: eight-week-old larvae fed with treatment 1, 2, 3 and 4; 1/10, 2/10, 3/10, 4/10: ten-week-old larvae fed with treatment 1, 2, 3 and 4.

**Table 4:** Summarized nutrient intake, mean and standard deviation (in mg/100 mg) of mealworm larvae at different ages (six-, eight- and ten-weeks-old, *n* = 4) and the sum of all groups (*n* = 12).

Nutrient (g/100 g)	Larval Age (in Weeks)			Sum
	6	8	10	
Carbohydrates	7.06 ± 0.22	20.05 ± 0.54	47.50 ± 1.37	**72.45 ± 2.12**
Protein	1.70 ± 0.08	6.90 ± 0.38	19.69 ± 0.44	**27.84 ± 0.90**
Lipids	0.61 ± 0.02	2.26 ± 0.11	5.71 ± 0.21	**8.46 ± 0.34**
Fiber	1.68 ± 0.08	5.98 ± 0.28	14.55 ± 0.62	**21.96 ± 0.98**
Ash (minerals)	0.43 ± 0.03	1.05 ± 0.07	3.79 ± 0.23	**5.23 ± 0.33**

## Data Availability

The data presented in this study are available on request from the corresponding author.

## Supplementary Materials

The following supporting information can be downloaded at: https://www.mdpi.com/article/10.3390/insects13070657/s1, Table S1: Nutritional composition (as specified by the manufacturer) of substrates on a fresh weight (FW) basis (%) used for *Tenebrio molitor* diets.; Table S2: Consumption (g dry weight) of all substrates eaten by *T. molitor* larvae with different ages (six-, eight- and ten-weeks-old) in four self-selection treatment combinations with eight choices; mean ± standard deviation; *n* = 3.
