# Supplementary material for: Self-Selection of Feeding Substrates by Tenebrio molitor Larvae of Different Ages to Determine Optimal Macronutrient Intake and the Influence on Larval Growth and Protein Content
Source: Insects. 2022 Jul 21;13(7):657. doi: 10.3390/insects13070657 (PMC9323919; doi:10.3390/insects13070657)
Supplement: Supplementary file 1 [file insects-13-00657-s001.zip › insects-1816745-supplementary.pdf]

Supplementary material

Self-Selection of Feeding Substrates by *Tenebrio molitor* Larvae of Different Ages to Determine Optimal Macronutrient Intake and the Influence on Larval Growth and Protein Content

Nina Kröncke [1,*] and Rainer Benning [1]

[1] Institute of Food Technology and Bioprocess Engineering, University of Applied Sciences Bremerhaven, An der Karlstadt 8, 27568 Bremerhaven, Germany, nkroencke@hs-bremerhaven.de (N.K.); rbenning@hs-bremerhaven.de (R.B.)

* Correspondence: nkroencke@hs-bremerhaven.de; Tel.: +49-(0)-471-4823-269

Table S1. Nutritional composition (as specified by the manufacturer) of substrates on a fresh weight (FW) basis (%) used for *Tenebrio molitor* diets.

Substrate	Moisture (%)	Protein (% FW)	Fat (% FW)	Carbohydrates (% FW)	Fiber (% FW)	Ash (% FW)	Manufacturer
Apricot kernel flour	8.6	36.0	10.1	9.8	34.2	1.3	Grapoila, Virgin Oil Press KFt., Budapest, Hungary
Beet pulp (dry)	13.9	8.7	0.9	54.2	17.8	4.5	Nordzucker AG, Braunschweig, Germany
Brewer's yeast	10.2	46.0	2.0	32.8	1.0	8.0	Leiber GmbH, Bramsche, Germany
Chokeberry pomace	7.7	8.0	4.9	56.5	20.8	2.1	Holger Senger Vertrieb von Naturrohstoffen e. K., Dransfeld, Germany
Coconut flour	8.5	16.0	27.0	38.0	8.7	1.8	Fischmix, Iserlohn, Germany
Fava bean hulls	9.8	4.2	2.3	28.1	50.4	5.2	Schierbecker Handels GmbH & Co. KG, Felde, Germany
Flaxseed flour	7.9	22.9	33.0	25.0	6.6	4.6	Fischmix, Iserlohn, Germany
Flaxseed pomace	8.5	27.4	15.3	8.0	34.6	6.2	Demeterhof Schwab GmbH & Co. KG, Windsbach, Germany
Grape seed flour	5.2	10.5	4.6	20.0	59.0	0.7	Schoefer Naturprodukte, Schwerin, Germany
Lupine flour	5.6	43.0	12.0	10.0	28.0	1.4	Natura-Werk Gebr. Hiller GmbH & Co. KG, Hannover, Germany
Maize hulls	10.5	8.1	4.9	64.0	10.8	1.7	Nordgetreide GmbH & Co. KG, Lübeck, Germany
Milk thistle flour	9.5	22.3	7.2	3.6	46.6	10.8	Grapoila, Virgin Oil Press KFt., Budapest, Hungary
Mustard flour	8.6	36.6	12.6	18.8	23.0	0.4	Wohltuer by suralogics GmbH, Dörfles-Esbach, Germany
Oat bran	9.8	14.0	7.8	50.0	14.0	4.4	Demeterhof Schwab GmbH & Co. KG, Windsbach, Germany
Oat flakes	9.4	13.5	7.0	58.7	10.0	1.4	Gut & Günstig, Edeka AG & Co. KG, Hamburg, Germany
Pea protein flour	2.7	80.0	8.0	4.9	4.2	0.2	Raab Vitalfood GmbH, Rohrbach, Germany
Potato flakes	9.2	8.3	0.5	76.2	4.6	1.2	Frießinger GmbH & Co. KG, Kirchberg/Murr, Germany
Psyllium seed husks	9.0	3.8	2.8	0.0	84.1	0.3	Müfagro Naturkosthandel GmbH, Gomadingen, Germany
Rapeseed cake meal	9.7	28.5	15.0	29.9	11.0	5.9	Fischmix, Iserlohn, Germany

Raspberry seed press cake	4.8	8.6	6.0	28.2	50.8	1.6	Schierbecker Handels GmbH & Co. KG, Felde, Germany
Rice flour	11.9	6.5	1.0	79.0	0.5	1.1	Frießinger GmbH & Co. KG, Kirchberg/Murr, Germany
Rice protein flour	3.9	80.0	2.9	9.6	3.3	0.3	Raab Vitalfood GmbH, Rohrbach, Germany
Tiger nut flour	7.5	3.8	26.2	39.8	20.8	1.9	Himmelbauer GmbH, Aschaffenburg, Germany
Wheat bran	12.0	14.9	4.7	45.0	17.7	5.7	Aurora Mühlen GmbH, Hamburg, Germany
Wheat flour	11.5	10.8	1.0	71.8	4.5	0.4	Frießinger GmbH & Co. KG, Kirchberg/Murr, Germany

Table S2. Consumption (g dry weight) of all substrates eaten by *T. molitor* larvae at different ages (six-, eight- and ten-weeks-old) in four self-selection treatment combinations with eight choices; mean ± standard deviation; $n = 3$.

**Substrate**	**Group**											
	**1/6**	**1/8**	**1/10**	**2/6**	**2/8**	**2/10**	**3/6**	**3/8**	**3/10**	**4/6**	**4/8**	**4/10**
Apricot kernel flour										0.00 ± 0.00	0.00 ± 0.00	0.00 ± 0.00
Beet pulp (dry)										0.00 ± 0.00	0.00 ± 0.00	0.20 ± 0.26
Brewer's yeast				0.00 ± 0.00	0.15 ± 0.19	0.23 ± 0.27	0.00 ± 0.00	0.05 ± 0.07	0.04 ± 0.05			
Chokeberry pomace										0.00 ± 0.00	0.00 ± 0.00	0.00 ± 0.00
Coconut flour							0.01 ± 0.02	0.00 ± 0.00	0.04 ± 0.01			
Fava bean hulls	0.00 ± 0.00	0.00 ± 0.00	0.15 ± 0.11									
Flaxseed flour				0.00 ± 0.00	0.00 ± 0.00	0.00 ± 0.00						
Flaxseed pomace										0.00 ± 0.00	0.00 ± 0.00	0.00 ± 0.00
Grape seed flour				0.00 ± 0.00	0.00 ± 0.00	0.00 ± 0.00						
Lupine flour	0.00 ± 0.00	0.67 ± 0.29	1.05 ± 0.38	0.00 ± 0.00	0.00 ± 0.00	0.35 ± 0.32				0.05 ± 0.00	0.32 ± 0.25	0.59 ± 0.22
Maize hulls				0.72 ± 0.09	1.40 ± 0.08	1.65 ± 0.02						
Milk thistle flour							0.01 ± 0.01	0.17 ± 0.08	1.04 ± 0.56			
Mustard flour							0.00 ± 0.00	0.00 ± 0.00	0.00 ± 0.00			
Oat bran							0.00 ± 0.00	1.28 ± 0.99	3.82 ± 0.89	0.08 ± 0.01	0.86 ± 0.79	2.57 ± 0.36
Oat flakes							0.47 ± 0.09	1.03 ± 0.97	1.72 ± 0.51	0.26 ± 0.01	0.29 ± 0.21	2.20 ± 0.74
Pea protein flour	0.00 ± 0.00	0.00 ± 0.00	0.00 ± 0.00									
Potato flakes	0.00 ± 0.00	0.16 ± 0.11	1.33 ± 0.11									
Psyllium seed husks							0.00 ± 0.00	0.00 ± 0.00	0.00 ± 0.00			
Rapeseed cake meal	0.00 ± 0.00	0.00 ± 0.00	0.00 ± 0.00									
Raspberry seed press cake				0.00 ± 0.00	0.00 ± 0.00	0.13 ± 0.09						
Rice flour	0.02 ± 0.03	0.86 ± 0.12	2.46 ± 0.28									
Rice protein flour				0.00 ± 0.00	0.22 ± 0.12	2.23 ± 0.50						
Tiger nut flour	0.00 ± 0.00	0.00 ± 0.00	0.39 ± 0.36									
Wheat bran	1.12 ± 0.08	2.51 ± 0.14	4.48 ± 1.62	0.39 ± 0.28	1.72 ± 0.32	3.05 ± 0.64	0.46 ± 0.12	1.48 ± 0.25	3.19 ± 0.82			
Wheat flour										0.72 ± 0.03	0.52 ± 0.40	0.85 ± 0.48
**Total consumption**	**1.14 ± 0.11**	**4.20 ± 0.66**	**9.86 ± 2.86**	**1.11 ± 0.37**	**3.49 ± 0.71**	**7.64 ± 1.52**	**0.95 ± 0.24**	**4.01 ± 2.36**	**9.85 ± 2.84**	**1.12 ± 0.05**	**1.99 ± 1.65**	**6.41 ± 2.06**

1/6, 2/6, 3/6, 4/6: six weeks old larvae fed with treatment 1, 2, 3 and 4; 1/8, 2/8, 3/8, 4/8: eight weeks old larvae fed with treatment 1, 2, 3 and 4; 1/10, 2/10, 3/10, 4/10: ten weeks old larvae fed with treatment 1, 2, 3 and 4.
